# Optical Camera-Based Integrated Sensing and Communication for V2X Applications: Model and Optimization

**DOI:** 10.3390/s25227061

**Published:** 2025-11-19

**Authors:** Ke Dong, Wenying Cao, Mingjun Wang

**Affiliations:** 1 School of Automation and Information Engineering, Xi’an University of Technology, Xi’an 710049, China; 3210432028@stu.xaut.edu.cn (W.C.); wangmingjun@xaut.edu.cn (M.W.); 2 Xi’an Key Laboratory of Wireless Optical Communication and Network Research, Xi’an 710049, China

**Keywords:** visible light communication, optical camera communication, integrated sensing and communication, OC-ISAC, vehicle-to-everything communication, channel model, exposure effect

## Abstract

An optical camera-based integrated sensing and communication (OC-ISAC) system model is proposed to address the intrinsic requirements of vehicular-to-everything (V2X) applications in complex outdoor environments. The model enables the coexistence and potential mutual enhancement of environmental sensing and data transmission within the visible light spectrum. It characterizes the OC-ISAC channel by modeling how light, either actively emitted for communication or passively reflected from the environment, originating from any voxel in three-dimensional space, propagates to the image sensor and contributes to the observed pixel values. This framework is leveraged to systematically analyze the impact of camera imaging parameters, particularly exposure time, on the joint performance of sensing and communication. To address the resulting trade-off, we develop an analytically tractable suboptimal algorithm that determines a near-optimal exposure time in closed form. Compared with the exhaustive numerical search for the global optimum, the suboptimal algorithm reduces computational complexity from O(N) to O(1), while introducing only a modest average normalized deviation of 5.71%. Both theoretical analysis and experimental results confirm that, in high-speed communication or mobile sensing scenarios, careful selection of exposure time and explicit compensation for the camera’s low-pass filtering effect in receiver design are essential to achieving optimal dual-functional performance.

## 1. Introduction

In vehicular communication networks, optical camera communication (OCC) has emerged as a promising alternative to traditional radio-frequency systems for vehicle-to-everything (V2X) connectivity [1,2]. By leveraging the widespread deployment of LED light sources and vehicle-mounted cameras, OCC enables low-cost, lightweight transceivers operating over the unlicensed optical spectrum. Its inherently limited coverage further enhances communication security by reducing the likelihood of eavesdropping. Despite these advantages, applying OCC to V2X scenarios encounters two categories of challenges. First, the outdoor vehicular environment is highly dynamic, with rapidly changing propagation conditions, fluctuating illumination, and strong natural and artificial light interference, leading to severe attenuation and multipath distortion [3]. Second, using a camera as the communication receiver introduces inherent non-idealities from the imaging mechanism—such as exposure-dependent integration, low frame rates, sensor nonlinearities, and shutter constraints—that significantly distort the recovered waveform [4]. These environmental and sensor-induced impairments jointly shape the received optical signal, thereby tightly coupling communication and sensing behaviors.

To address these limitations, intensive research has been conducted in multiple dimensions. From a channel characterization perspective, studies have focused on modeling the propagation environment [5], camera imaging models [6], and exposure-effect analysis [7]. In terms of communication mechanisms, advancements include efficient modulation and coding schemes [8,9,10,11], resource multiplexing strategies [12,13], channel equalization techniques [14,15,16], and region-of-interest (RoI) detection and tracking approaches [17,18]. Performance evaluation studies have investigated how bit error rate (BER) [19] and channel capacity [20] are affected by exposure effects and link distance [21]. On the standardization front, several physical-layer techniques for OCC have been specified within the amended IEEE 802.15.7 standard [22]. In addition, studies have demonstrated that OCC can be integrated with object detection [23] and localization techniques [24,25], proving the coexistence and mutual enhancement of communication and sensing.

The dual role of the camera as a sensor and receiver makes OCC similar to integrated sensing and communication (ISAC) in radio frequency vehicular communication [26,27,28], which explores service coexistence, functional cooperation, and network reciprocity. However, the conclusions for ISAC cannot be directly applied to optical camera-based ISAC (OC-ISAC) because of the differences in system architecture. In recent years, integrated optical communication and sensing (O-ISAC) has gained significant attention in fields such as fiber-optic communication, free-space optical (FSO) communication, and visible light communication (VLC) [29,30,31,32,33,34].

Although OCC has been widely studied, existing work typically addresses either its communication challenges or the use of communication signals to support sensing, without situating OCC within a unified ISAC framework. Current O-ISAC studies are further limited to indoor or low-dynamic settings and do not account for the rapidly changing illumination and mobility inherent to outdoor V2X scenarios. As a result, no existing approach leverages the camera’s optical imaging mechanism to establish a unified model that jointly analyzes and optimizes sensing and communication, nor to quantify the potential performance gains of optical camera-based integrated sensing and communication (OC-ISAC) for vehicular applications.

This paper introduces a novel OC-ISAC system model for outdoor V2X communication that accounts for the camera’s dual role in both imaging and communication. An integrated channel model is proposed accordingly, incorporating voxel-dependent reflection and luminous factors to characterize pixel value variations caused by transceiver mobility and LED flicker. This model enables performance analysis of both environmental sensing and data communication. Additionally, an optimization problem is formulated for camera exposure time, and a suboptimal yet analytically tractable solution is derived to balance the trade-offs between the two functionalities.

The rest of this paper is organized as follows. Section 2 introduces the proposed OC-ISAC system architecture and develops an integrated channel model for sensing and communication based on the camera’s imaging mechanism. Section 3 assesses the impact of the integrated channel on the performance of both sensing and communication. Section 4 addresses optimizing the camera’s exposure time to maximize the average signal-to-noise ratio (SNR) for communication and sensing. Section 5 presents numerical and simulation results to validate the theoretical analysis. Finally, Section 6 concludes the paper and suggests potential directions for future research.

## 2. System Model

### 2.1. OC-ISAC Architecture

**Figure 1:**
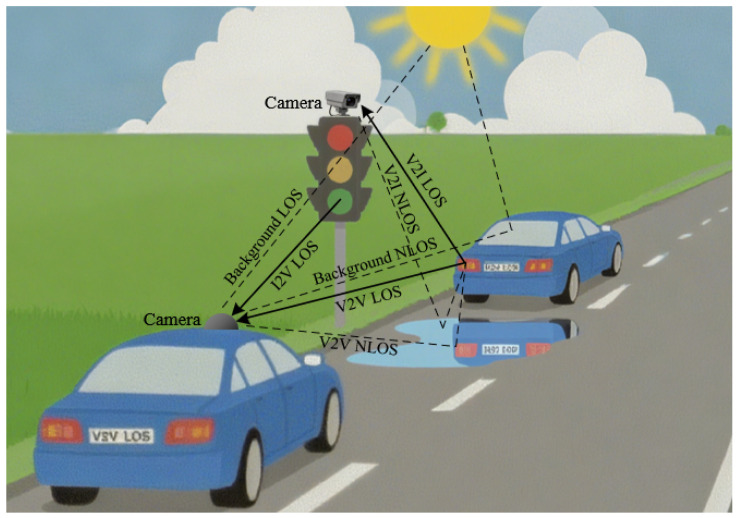
Illustration of a typical OCC-based V2X communication scenario. V2V: vehicle’s head/tail lights to onboard camera. V2I: vehicle’s head/tail lights to surveillance camera. I2V: traffic lights to onboard camera.

Figure 1 illustrates a typical V2X communication scenario based on OCC. While driving, vehicles exchange information with other vehicles (V2V) or infrastructure (V2I/I2V) to enhance traffic efficiency and safety. At the transmitter side, LEDs embedded in headlights, taillights, or traffic signals modulate the data. On the receiver side, onboard and surveillance cameras capture the optical signals to retrieve the transmitted information. Before that, in dynamic outdoor environments, the receivers must detect, identify, and track communication sources despite significant interference from line-of-sight (LOS) and non-line-of-sight (NLOS) paths. This highlights the importance of integrating sensing and communication. Therefore, establishing an OC-ISAC system is crucial for ensuring reliable V2X communications in complex outdoor conditions.

**Figure 2:**
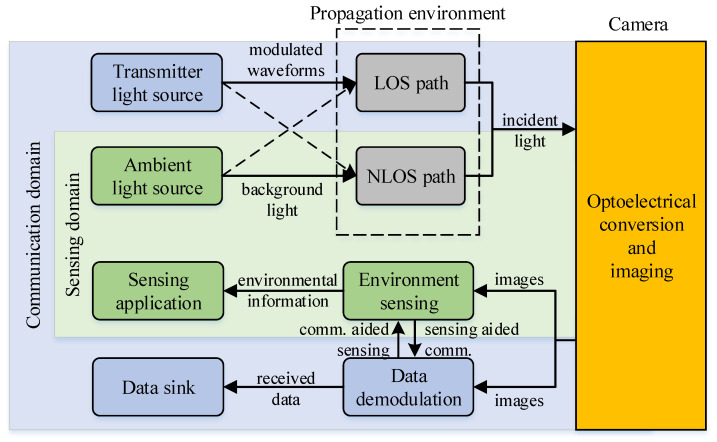
Architecture of the OC-ISAC system, which simultaneously enables communication (blue area) and sensing (green area) with a shared camera. Data-modulated communication light and environment-reflected sensing light, propagating via LOS and/or NLOS paths, are captured by a camera and converted into digital pixel values through photoelectric detection. The spatial–temporal distribution of these pixel values is jointly exploited by the communication and sensing receivers to extract data symbols and environmental information, respectively. Moreover, the two functions mutually assist each other, enabling collaborative communication and sensing.

Figure 2 illustrates the architecture of the OC-ISAC system, which leverages the optoelectronic conversion capabilities of image sensors to enable simultaneous environmental perception and data communication. The system categorizes light sources into two types: communication light and sensing light. Communication light is emitted by vehicle and infrastructure LEDs and transmitted through intensity modulation. In contrast, sensing light, which includes sunlight, moonlight, and artificial illumination, provides environmental information through reflection.

This paper examines a typical scenario in which communication light travels along direct line-of-sight paths to optimize the SNR. In contrast, sensing light arrives through NLOS paths, often via reflection. In practice, both communication and sensing light can reach the camera through LOS and NLOS paths. For example, wet road reflections or backlighting make these signals deviate from their expected paths, which complicates reliable sensing and communication.

At the receiver, the optical camera serves as the system core. Converting incident light into digital image sequences generates pixel sets that simultaneously encode data and environmental information. The communication domain demodulates the transmitted data according to pixel spatiotemporal patterns and outputs payloads to higher layers. In parallel, the sensing domain extracts features from image sequences, enabling scene understanding and providing standardized metrics for sensing applications.

In this manner, OC-ISAC achieves the dual function of sensing and communication through a shared hardware infrastructure (LEDs and cameras). More importantly, the two functions are mutually enhanced. For instance, reliable communication links depend on real-time light-source tracking, essentially a sensing task, while camera-based VLC positioning benefits from communication-derived source information, significantly improving localization accuracy.

### 2.2. List of Symbols

For clarity and convenience, the key symbols and definitions employed in this work are listed in Table 1.

### 2.3. Voxel-to-Pixel Channel Model

The OCC-based V2X application scenario in Figure 1 and the OC-ISAC architecture in Figure 2 highlight that the communication and sensing functions rely on the light propagated through a complex three-dimensional environment and captured by a camera-based receiver. Such characteristics cannot be sufficiently described by conventional wireless channel models or standard optical LOS formulations. To capture the spatially distributed nature of light interaction in OC-ISAC, it is necessary to introduce the concept of a voxel, which represents a small volumetric element in the 3D environment. Each voxel acts as an elementary optical contributor whose reflectance or self-emission influences one or more pixels depending on its geometric projection and distance to the camera. Modeling the channel via a voxel-to-pixel mapping explicitly describes how the environmental structure transforms emitted or reflected light into a pixel-wise intensity distribution. This modeling approach allows the contributions of voxels to be integrated into the camera exposure process, thereby linking the physical propagation, spatial geometry, and temporal integration into a unified analytical form suitable for ISAC analysis.

**Figure 3:**
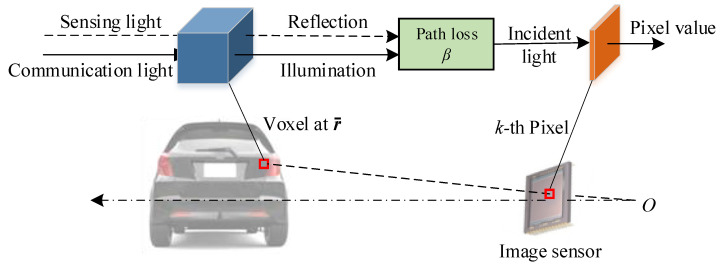
Illustration of the voxel-to-pixel channel model for OC-ISAC. The *k*-th pixel value corresponding to a vehicle’s taillights in the camera output image is determined by the sum of the light intensities incident on the pixels, which results from the propagation path loss β of two components originating from a voxel on the taillight at the position $\vec{r}$ in 3-D space: (1) illumination used as communication light for data modulation, and (2) reflection of ambient light used as sensing light for acquiring environmental information.

Figure 3 illustrates the proposed voxel-to-pixel (VP) channel model for OC-ISAC. For a given pixel *k* on the 2-D image sensor, we can determine its corresponding voxel at a position vector $\vec{r}$ in 3-D space using the pinhole camera geometry, which relies on the camera’s parameters and the physical environment. This process establishes a mapping from voxel to pixel. The information represented by the pixel value is directly related to the optical properties of the corresponding voxel, particularly how it interacts with communication and sensing light. Utilizing this voxel-to-pixel mapping, we systematically describe the OC-ISAC channel model from three perspectives: the communication channel, the sensing channel, and the pixel value generation process.

#### 2.3.1. Communication Channel

When a voxel is part of a communication light source (e.g., a car’s headlamp or taillight), it emits light to a corresponding pixel. In an intensity modulation and direct detection (IM/DD) system, the transmitter modulates data by varying the light intensity over time. The voxel’s optical property is the product of a time-varying luminous factor (LF), $\eta \left(\vec{r},t\right)\in \left[0,1\right]$, and the source’s maximum luminous intensity, $I_{c}$. The modulation scheme defines LF’s temporal behavior. For example, in On-Off Keying (OOK), LF is binary (0 or 1), while in continuous wave modulation, LF can vary continuously between 0 and 1. Thus, LF’s temporal behavior models the communication method in the OC-ISAC system. The received luminous intensity at pixel *k* from the voxel, assuming distance *d*, is expressed as follows:

(1) $$i_{k,c}\left(t\right)=\beta \left(d\right)\cdot I_{c}\cdot \eta \left(\vec{r},t\right)$$

where β(d) represents the path loss factor over the distance *d*.

#### 2.3.2. Sensing Channel

When a voxel is illuminated by light, it reflects off its surface and projects onto the camera’s image sensor, creating a second incident light beam. Assuming constant ambient light intensity, $I_{e}$, the voxel’s reflectance determines the light intensity at a pixel. As the voxel corresponding to a pixel changes over time due to relative motion, the reflectance varies. The reflectance of voxel $\vec{r}$ at pixel *k* can be modeled as a time-varying reflection factor (RF) $\xi \left(\vec{r},t\right)\in \left[0,1\right]$. RF’s temporal variation depends on relative motion, speed, and surrounding voxel reflectance. Thus, the luminous intensity of ambient light projected onto the pixel after reflection from a voxel at a distance *d* is expressed as follows:

(2) $$i_{k,e}\left(t\right)=\beta \left(d\right)\cdot I_{e}\cdot \xi \left(\vec{r},t\right).$$

In practical scenarios, a given voxel corresponding to a pixel may simultaneously function as both a communication light source and a sensing object, which means it can both emit and reflect light. Therefore, the total luminous intensity incident on a pixel is the sum of the two light beams:

(3) $$i_{k}\left(t\right)=i_{k,c}\left(t\right)+i_{k,e}\left(t\right)=\beta \left(d\right)\left[I_{c}\eta \left(\vec{r},t\right)+I_{e}\xi \left(\vec{r},t\right)\right].$$

#### 2.3.3. Pixel Value

**Figure 4:**
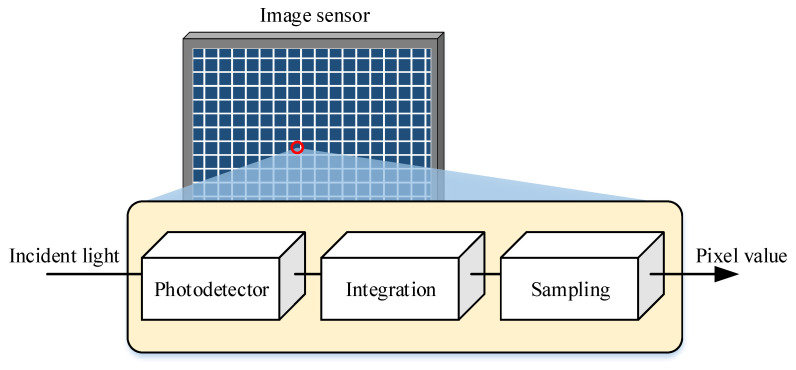
Schematic diagram of the photoelectric conversion process in pixels of image sensors.

An image sensor converts the optical intensity incident on each pixel into a digital pixel value, which is organized in a two-dimensional matrix to form an image. The imaging process is governed by a precisely controlled exposure mechanism. As illustrated in Figure 4, a chain of “photodetector, integration, and sampling” summarizes the conversion from incident light to pixel value output for each pixel in the image sensor.

Each pixel integrates the incident optical power during exposure, which is controlled by the shutter. The photodetector generates a photocurrent that accumulates over the exposure duration $T_{e}$. The accumulated current is converted into a voltage through a trans-impedance amplifier (TIA) and subsequently sampled at the end of the exposure. The digital value of a pixel is obtained after saturation and analog-to-digital conversion (ADC). For analytical tractability, the effects of ADC quantization and saturation are neglected. Therefore, the output of the *k*-th pixel can then be approximated as the accumulated photocurrent during $T_{e}$ as follows:

(4) $$v_{k}\left(t\right)=A\int_{t-T_{e}}^{t}i_{k}\left(\tau \right)d\tau +n\left(t\right),$$

where *A* represents the conversion gain of the image sensor, which accounts for the responsibility of the photodetector and the amplification factor of the trans-impedance amplifier [6], and n(t) represents additive white Gaussian noise (AWGN) with zero mean and power spectral density $N_{0}$. The digital pixel value is obtained by sampling $v_{k}\left(t\right)$ at the end of the exposure.

### 2.4. Signal Model

This section derives the output signal expressions for communication and sensing under given modulation, environmental, and imaging parameters.

#### 2.4.1. Communication Signal

Assuming a single-tone continuous wave modulation scheme (the derivation also holds for other schemes, e.g., OOK), the LF in Equation (1) is as follows:

(5) $$\eta \left(\vec{r},t\right)=a_{c}\left[\frac{1}{2}+\frac{1}{2}\cos\left(2\pi f_{c}t+\phi_{c}\right)\right]$$

where $a_{c}\in \left[0,1\right]$, $f_{c}>0$, and $\phi_{c}\in \left[0,2\pi \right]$ denote the carrier’s amplitude, frequency, and phase, respectively. The constant offset 1/2 ensures non-negativity of the intensity-modulated signal.

#### 2.4.2. Sensing Signal

Imaging maps object reflectance in 3-D space to pixel values in 2-D images, enabling environment sensing. Camera motion causes translational shifts in output images, and excessive speed introduces motion blur, degrading sensing quality. In the VP channel model, mobility alters voxel–pixel mapping, making pixel values fluctuate based on environmental complexity (i.e., reflectance differences between adjacent voxels) and motion speed. We use a simplified model to study temporal variations in pixel values as the camera perceives environments of different complexities during motion.

Suppose that the reflectance of the voxels in a given environment along the *x*-axis follows a cosine distribution:

(6) $$u\left(\vec{r}\right)=\frac{1}{2}+\frac{1}{2}\cos\left(2\pi f_{x}\vec{r}\right)$$

where $f_{x}$ is the spatial frequency characterizing scene complexity, the direct current (DC) component with a scaling factor 1/2 ensures $u\left(\vec{r}\right)\in \left[0,1\right]$. Although actual reflectance variations can be expressed as a Fourier series with infinite spatial frequencies in all directions, Equation (6) represents the simplest form, featuring a single frequency component $f_{x}$ along a specific direction *x*.

If the voxel moves uniformly along the *x*-axis with speed *v*, the reflectance evolves as a temporal modulation given by the following:

(7) $$\xi \left(\vec{r},t\right)=u\left(\vec{r}-vt\right)=\frac{1}{2}+\frac{1}{2}\cos\left(2\pi f_{x}vt-\phi_{x}\left(\vec{r}\right)\right)$$

where $\phi_{x}\left(\vec{r}\right)$ is an initial phase determined by voxel position. It implies that the incident light intensity oscillates at a frequency $f_{x}v$, i.e., the product of environment complexity and motion speed.

Substituting Equations (5) and (7) into Equation (4) yields the induced voltage after exposure:

(8) $$\begin{array}{cc} v_{k}\left(t\right)= & C_{c}+E_{c}\cos\left(2\pi f_{c}t+\phi -D_{c}\right)\\ \; & +C_{e}+E_{e}\cos\left(2\pi f_{x}vt+\phi_{x}\left(\vec{r}\right)-D_{e}\right)+n\left(t\right) \end{array}$$

where

(9) $$\begin{array}{cccc} C_{c} & =\frac{A\beta \left(d\right)a_{c}I_{c}T_{e}}{2}, & C_{e} & =\frac{A\beta \left(d\right)I_{e}T_{e}}{2}\\ E_{c} & =\frac{A\beta \left(d\right)a_{c}I_{c}\sin\left(\pi f_{c}T_{e}\right)}{2\pi f_{c}}, & E_{e} & =\frac{A\beta \left(d\right)I_{e}\sin\left(\pi f_{x}vT_{e}\right)}{2\pi f_{x}v}\\ D_{c} & =\pi fT_{e}, & D_{e} & =\pi f_{x}vT_{e}. \end{array}$$

The final pixel value is obtained following sampling, ADC, and saturation, governed by the shutter mechanism.

## 3. Performance Analysis

This section examines how the camera imaging mechanism affects OC-ISAC performance. Communication seeks to recover source fluctuations from pixel values to estimate the carrier and symbols, while sensing aims to recover voxel reflectance to infer the environment. However, pixel values deviate from incident light levels depending on imaging settings, which inevitably complicates parameter estimation. Thus, analyzing imaging parameters—especially exposure time—is essential for understanding and optimizing OC-ISAC systems.

### 3.1. Exposure Effect

From Equations (8) and (9), it can be observed that, in addition to amplitude attenuation caused by propagation distance and photoelectric conversion, the camera exposure effect also changes the signal structure. Specifically, when the incident light intensity varies as a cosine function, the induced voltage of a pixel retains a cosine with the same frequency, but subject to a frequency-dependent amplitude attenuation (e.g., $E_{c}$ and $E_{e}$) and additional phase shift (e.g., $D_{c}$ and $D_{e}$). Such a transmission characteristic reflects the low-pass filtering behavior of the pixel-level photoelectric conversion process. Given the exposure time $T_{e}$, the corresponding frequency response is as follows:

(10) $$H\left(f,T_{e}\right)=e^{-j\pi fT_{e}}\frac{\sin(\pi fT_{e})}{\pi f},$$

for $f\geq 0,T_{e}>0$. As a result, high-frequency components of the modulated signal are attenuated, and the captured image intensity can be expressed as a convolution of the incident signal with a rectangular window of width $T_{e}$. This temporal integration leads to waveform distortion, where the edges of square-wave modulation or high-speed flicker signals become smoothed, manifesting as amplitude compression and phase delay in the sampled optical signal. Therefore, the choice of $T_{e}$ directly determines the extent of this distortion and the achievable communication bandwidth in the OC-ISAC system.

**Figure 5:**
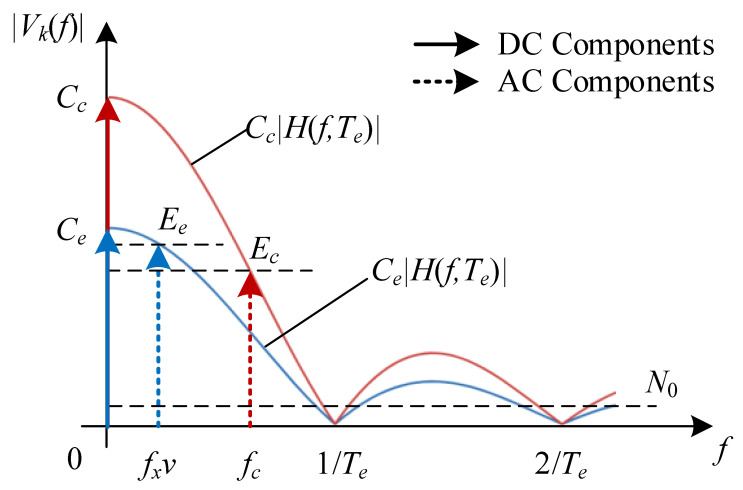
Frequency response of the induced voltage in communication and sensing signals, illustrating the low-pass filtering effect of camera exposure with duration $T_{e}$.

Figure 5 illustrates the amplitude–frequency characteristics of the induced voltage $v_{k}\left(t\right)$ for a pixel. The communication and sensing signals consist of DC and alternating current (AC) components. The DC components correspond to $C_{c}$ and $C_{e}$ in Equation (8), originating from the optical bias in the incident light, and carry no information. The AC components comprise two cosine functions with frequency $f_{c}$ and amplitude $E_{c}$ for communication, and frequency $f_{x}v$ and amplitude $E_{e}$ for sensing, respectively. According to Equations (9) and (10), the exposure effect of the camera attenuates these AC components following a Sinc-shaped frequency response as follows:

(11) $$E_{c}=C_{c}\left|H(f_{c},T_{e})\right|$$

and

(12) $$E_{e}=C_{e}\left|H(f_{x}v,T_{e})\right|.$$

### 3.2. Normalized Gains for Communication and Sensing

The SNR of the received signal is a key performance indicator for communication. As shown in Figure 5, the effective SNR for communication is defined as a ratio of the power spectrum density of the AC component and AWGN:

(13) $$\rho_{c}=\frac{E_{c}^{2}}{N_{0}}.$$

Given an average received SNR, i.e., ${\bar{\rho }}_{c}=C_{c}^{2}/N_{0}$, we have the following:

(14) $$\rho_{c}=\frac{E_{c}^{2}}{C_{c}^{2}}\cdot \frac{C_{c}^{2}}{N_{0}}=G_{c}^{2}{\bar{\rho }}_{c}$$

where $G_{c}=E_{c}/C_{c}$ is defined as the modulation gain, quantifying the relative attenuation of the information-bearing AC component with respect to the DC component under the exposure effect. From Equation (11), it follows that

(15) $$G_{c}=\left|H(f_{c},T_{e})\right|.$$

In sensing applications, image quality is often assessed using contrast gain, which is defined as the ratio of the maximum range of pixel values to the average pixel value. This metric indicates how visible features are against the background. A higher contrast gain enhances the detectability of important features. Therefore, evaluating the sensing performance in OC-ISAC involves analyzing the average contrast gain across all pixels. As indicated in Figure 5 and Equation (12), the contrast gain is as follows:

(16) $$G_{e}=\frac{E_{e}}{C_{e}}=\left|H(f_{x}v,T_{e})\right|.$$

The analysis shows that the camera’s exposure effect significantly impacts communication and sensing performance in OC-ISAC. Using cameras with different exposure times across various frequencies and mobility scenarios yields distinct trade-offs in performance between the two areas. It is noteworthy that, although this paper does not use bit error rate (BER) or mean square error (MSE) as performance indicators for communication and sensing services, respectively, we use the normalized gains to construct evaluation indicators and optimization problems, which have the same effect.

## 4. Optimization of the Camera’s Exposure Time

As noted, rapid flickering and transceiver motion cause temporal variations in pixel intensity. However, camera exposure’s low-pass filtering limits accurate reconstruction, degrading both communication and sensing performance, especially in high data rate or mobility scenarios, where exposure time limits system bandwidth, reducing reliability and accuracy. In practical applications, communication and sensing may have conflicting exposure time requirements. For example, a short exposure time is needed for high-speed communication, but it reduces image brightness and sensing quality. Extending the exposure to improve brightness blurs the communication signal. Therefore, it is crucial to optimize exposure time based on performance analysis.

### 4.1. Problem Formulation

In an outdoor V2X communication scenario, the objective of OC-ISAC performance optimization is to determine an appropriate camera exposure time $T_{e}$ such that the average performance of both communication and sensing is maximized:

(17) $${\overset{⌢}{T}}_{e}=\underset{T_{e}>0}{\arg\;\max}\{Y(T_{e})\}=\underset{T_{e}>0}{\arg\;\max}\{G_{c}+G_{e}\}.$$

Although the weights for combining sensing and communication services in the objective function can be adjusted based on specific scenarios; an equal weight strategy is employed here to suit a neutral situation.

By substituting Equations (10), (15) and (16) into Equation (17), the objective function is as follows:

(18) $$Y\left(T_{e}\right)=\left|\frac{\sin(\pi f_{c}T_{e})}{\pi f_{c}}\right|+\left|\frac{\sin(\pi f_{x}vT_{e})}{\pi f_{x}v}\right|.$$

**Figure 6:**
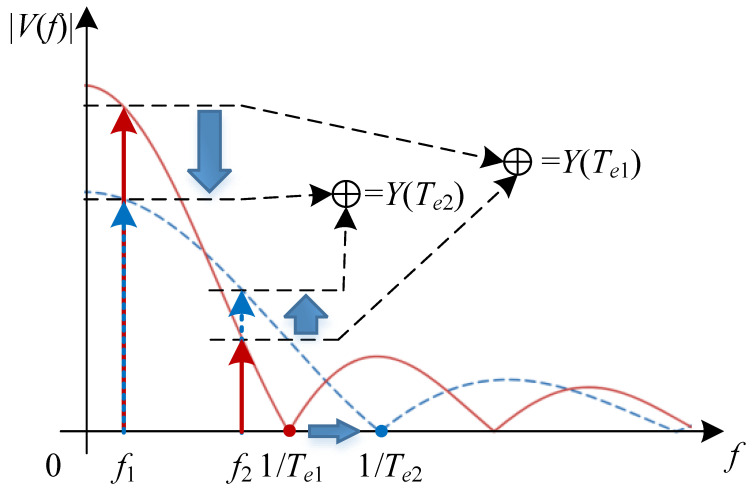
Variation trend of the optimization objective function with respect to exposure time.

As shown in Figure 6, changes in $T_{e}$ the shift of the maxima and zeros of the Sinc-shaped response, altering the effective amplitudes at $f_{c}$ and $f_{x}v$. Thus, solving the optimization problem involves finding the optimal main-lobe width $1/T_{e}$ that maximizes the sum of the vertical cut-line values at these frequencies.

### 4.2. Suboptimal Solution

The optimization problem formulated in Equation (17) is analytically intractable. While the globally optimal exposure time can be obtained via numerical search over a discretized grid within a feasible range—requiring O(N) computational complexity, where *N* denotes the number of candidate points—this approach is impractical for real-time vehicular applications. To address this, we propose a low-complexity suboptimal algorithm with O(1) complexity that analytically computes a near-optimal exposure time, effectively balancing communication and sensing performance.

The algorithm is summarized as follows.

1. **Input parameters:** Obtain the communication modulation frequency $f_{c}$ and the sensing frequency $f_{x}v$.
2. **Frequency classification:** Determine the higher and lower characteristic frequencies:
  (19) $$f_{\max}=\max\left(f_{c},f_{x}v\right),\;f_{\min}=\min\left(f_{c},f_{x}v\right).$$
3. **Compute the frequency ratio:** Evaluate the ratio
  (20) $$\varepsilon =\frac{f_{\max}}{f_{\min}},$$
  which reflects the disparity between the communication and sensing frequency components.
4. **Determine the side-lobe index:** Estimate the integer parameter γ indicating the number of side lobes between $f_{c}$ and $f_{x}v$ in the Sinc-shaped response:
  (21) $$\gamma =\max\;\left(\left\lfloor \frac{\varepsilon }{2}-0.5\right\rfloor ,0\right).$$
5. **Compute the suboptimal exposure time:** The exposure time that approximately maximizes the joint performance is given by the following:
  (22) $${\tilde{T}}_{e}=\frac{\gamma +0.5}{f_{\max}}.$$
6. **Output:** The obtained ${\tilde{T}}_{e}$ represents the suboptimal exposure time balancing communication reliability and sensing accuracy in the OC-ISAC system.

It is noteworthy that when $f_{c}\approx f_{x}v$ (i.e., ε≈1), the algorithm simplifies to the following:

(23) $${\tilde{T}}_{e}=\frac{1}{2f_{\max}},$$

corresponding to the main-lobe alignment condition. The algorithm’s computational complexity is O(1), making it well-suited for real-time camera control in vehicular scenarios where computational efficiency is often prioritized over achieving a mathematically perfect optimum.

## 5. Experiment and Results

An OCC testbed is developed using an electric turntable and an annular grating as the transmitter to validate the impact of camera exposure on flicker detection and motion-aware sensing. Additionally, numerical simulations are performed to demonstrate the effectiveness of the proposed exposure time optimization algorithm for OC-ISAC.

### 5.1. Experiment Setup

**Figure 7:**
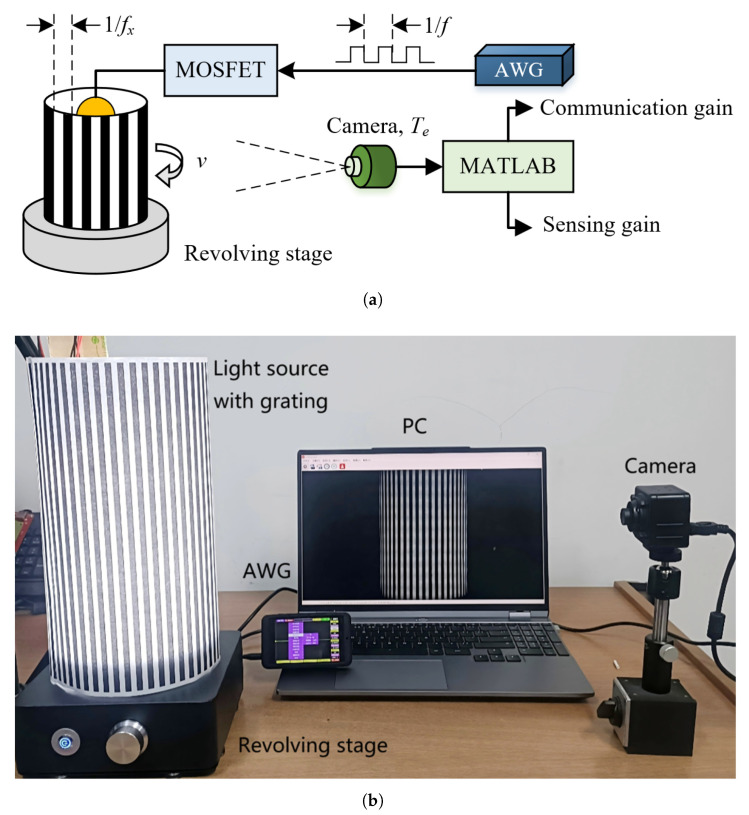
Schematic of the experimental setup. The signal generated by an arbitrary waveform generator (AWG) modulates the light intensity emitted by an LED light source placed inside an annular grating lampshade, through a MOSFET driver. The light passing through the grating is captured by a camera, producing a corresponding image sequence. These images are fed into MATLAB for analysis of the performance metrics of the communication and sensing functions. (**a**) schematic diagram and (**b**) photograph of the prototype.

The setup schematic and photograph are shown in Figure 7. The transmitter uses an arbitrary waveform generator (AWG) to generate square-wave signals that modulate a 3 W LED light source via a metal-oxide-semiconductor field-effect transistor (MOSFET) driver, emulating communication beacons at different modulation rates ($f_{c}$). The annular grating lampshades with different slot widths, mounted on an electric turntable, are used to simulate different spatial complexities ($f_{x}$). When the turntable rotates at various speeds (*v*), the sensing signals are thus produced. In addition, a rolling-shutter camera with configurable exposure time at the receiver is about 30 cm away from the source to capture video clips. The recorded image frames are extracted and processed in MATLAB 2021a for frequency-domain analysis, yielding normalized gains for both communication and sensing functions.

### 5.2. Methodology

**Figure 8:**
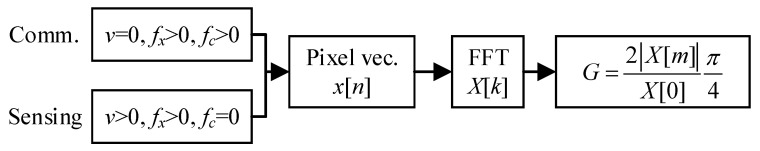
Flowchart of the signal processing chain for frequency-domain analysis in the receiver.

The experiments evaluate the signal gain of light-intensity modulations caused by source flicker and environmental motion under camera exposure, using frequency-domain analysis for both communication and sensing. Experimental parameters and the analysis flowchart are summarized in Table 2 and Figure 8.

The grating was kept stationary in the communication scenario while the light source was modulated at different frequencies. Conversely, a constant light source was used for the sensing scenario, and the grating was rotated at a constant angular speed, resulting in a linear speed of $v=\pi D_{r}N_{r}/60$.

As shown in Figure 8, the experiment utilizes the aforementioned setup under two initial conditions—(i) a flickering light source with a stationary grating, and (ii) an always-ON light source with a rotating grating—to evaluate the performance metrics of communication and sensing tasks, denoted as $G_{c}$ and $G_{e}$, respectively, through the following steps.

1. **Input parameters:** Confirm the parameter sets of {$v=0,f_{x}>0,f_{c}>0$} and {$v>0,f_{x}>0,f_{c}=0$} for communication and sensing scenarios, respectively.
2. **Pixel vector:** In each scenario, select an arbitrary column of pixels from the output image (in the sensing scenario, choose a column that can be transmitted through the grating) to obtain the pixel value vector, {x(n)} for x(n)∈[0,255] and n∈[0,N−1].
3. **Fast Fourier Transformation (FFT):** Compute an *N*-point FFT of x(n) to obtain its frequency spectrum vector, X(k)=F(x(n)) for k=0,1,...,N−1.
4. **Normalized gains:** Compute the normalized gains by the following:
  (24) $$G=\frac{2|X(m)|}{X(0)}\frac{\pi }{4}$$
  where *m* is the index of the frequency component in DFT caused by data modulation and mobility, which is determined by the following:
  (25) $$m=\left\{\begin{array}{cc} \left\lfloor NT_{r}f_{c}\right\rfloor & ,\mathrm{for}\;\mathrm{communication}\\ \left\lfloor NT_{r}f_{x}v\right\rfloor & ,\mathrm{for}\;\mathrm{sensing} \end{array}\right.$$

To simplify the experimental implementation, binary square-wave signals rather than sinusoidal signals were employed to control both temporal light intensity and spatial environmental variations. As a square wave can be represented as a superposition of a fundamental frequency component $f_{1}$ and its odd harmonics:

(26) $$\mathrm{squ}\left(t\right)=\frac{4}{\pi }\sum_{n=1,3,5,...}\frac{1}{n}\sin\left(2\pi nf_{1}t\right),$$

The measurement is normalized by the coefficient of the fundamental frequency component (4/π) when calculating the single-frequency signal gain from the square wave measurements (as shown in Figure 8).

### 5.3. Normalized Gains for Communication and Sensing

**Figure 9:**
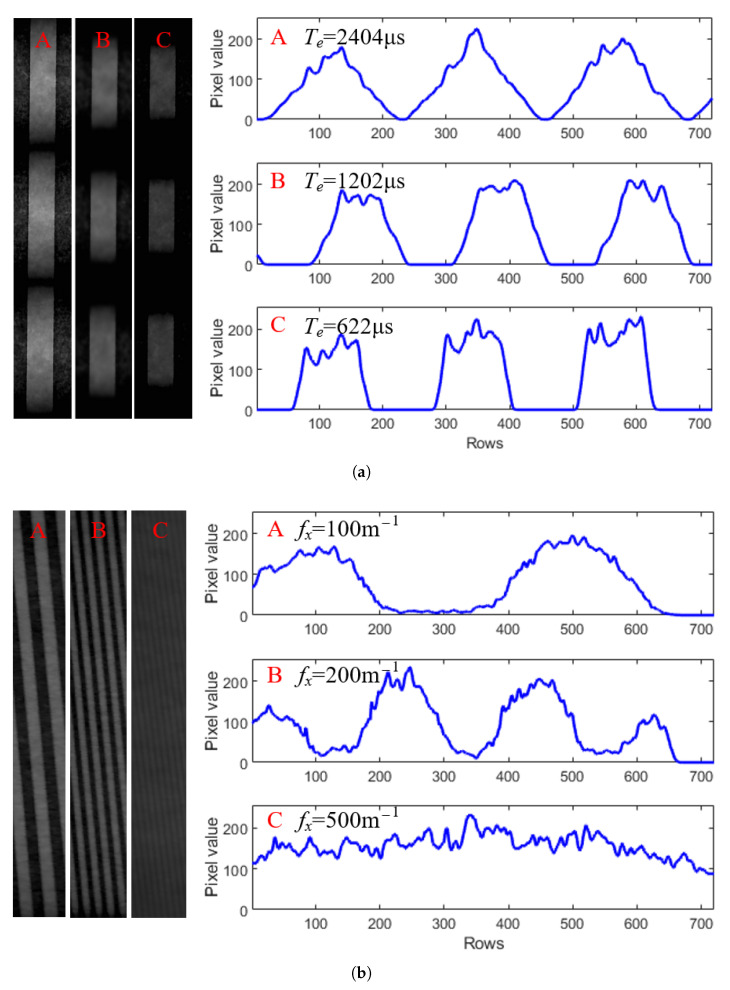
Acquired pixel values from the captured images in communication (**a**) and sensing (**b**) scenarios. The communication used a $f_{c}=200$ Hz flickering light source with a stationary turntable and the camera’s exposure times of (A) 2404 μs, (B) 1202 μs, and (C) 622 μs, while the sensing used a constant light source and a turntable with different grating slots rotating at an equivalent linear speed of v=1.75 m/s with environmental complexities of (A) 100 $m^{-1}$, (B) 200 $m^{-1}$, and (C) 500 $m^{-1}$.

Figure 9 shows the captured images and pixel value distributions for both communication and sensing scenarios. In the communication scenario (Figure 9a), the turntable is stationary, and the light source flickers at $f_{c}=200$ Hz, with videos recorded at three exposure times, i.e., (A) $T_{e}=2401$ μs, (B) $T_{e}=1202$ μs, and (C) $T_{e}=622$ μs. In the sensing scenario (Figure 9b), the light source is constant, and the turntable rotates at 215 RPM (1.75 m/s) with three environmental complexity (or grating widths), i.e., (A) $f_{x}=100\;m^{-1}$, (B) $f_{x}=200\;m^{-1}$, and (C) $f_{x}=500\;m^{-1}$, with videos recorded at a fixed exposure time $T_{e}=1202$ μs.

The periodic pixel value variations in both cases show that the flickering light source and environmental motion cause periodic changes in incident light intensity. This is due to the rolling shutter mechanism, which converts temporal variations into a spatial pattern along pixel columns. The camera’s exposure acts as a low-pass filter, affecting both communication and sensing performance. Therefore, this effect must be considered to reconstruct the source state and environmental information accurately.

Using the frequency-domain analysis in Figure 8, we determined the normalized signal gain from square waves, reflecting the gains of a single-frequency signal after the camera’s exposure effect. We recorded approximately one minute of video under each of two experimental configurations—communication and sensing—using a camera with exposure times of 4981 μs, 2404 μs, 1202 μs, and 622 μs, respectively. For each configuration, we processed approximately 1800 image frames for each exposure time using the method illustrated in Figure 8 to compute the normalized gains, i.e., $G_{c}$ and $G_{e}$, and reported their average values.

**Figure 10:**
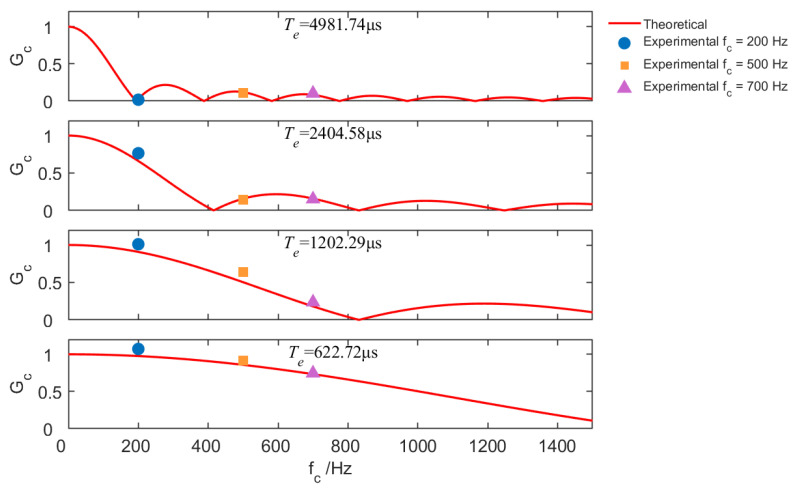
Normalized channel gain in the communication scenario, with varying data rates ($f_{c}$) and camera exposure times ($T_{e}$).

Figure 10 shows the averaged normalized gains measured in the communication scenario by transmitting square waves with $f_{c}$ equal to 200 Hz, 500 Hz, and 700 Hz. The results show a decrease in signal gain as frequency increases, consistent with theoretical analysis. It indicates that exposure time is a critical parameter that influences the distortion introduced by the camera in response to incident optical signals. In fact, a longer exposure time results in a lower cutoff frequency of the low-pass filtering effect, which reduces the bandwidth of the OCC channel and consequently limits the effective data transmission rate.

**Figure 11:**
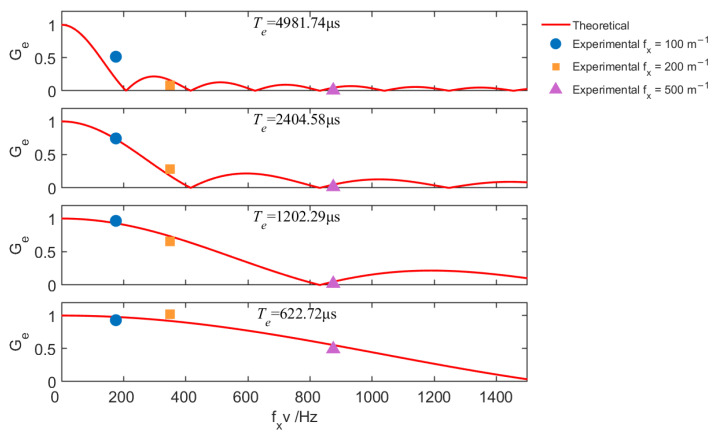
Normalized channel gain in the sensing scenario, with varying environmental complexities ($f_{x}$) and camera exposure times ($T_{e}$). The motion speed was held constant at v=1.75 m/s.

Figure 11 shows the averaged normalized gains for the sensing scenario at different exposure times, with gratings simulating environments of spatial complexities 100 $m^{-1}$, 200 $m^{-1}$, and 500 $m^{-1}$, while the turntable rotates at a linear speed of 1.75 m/s. The gain behavior in sensing is analogous to communication, where $f_{x}v$ acts as a “sensing frequency”. Under a fixed exposure time, faster relative motion between the transceiver and the environment—or sensing in a more complex scene—leads to a reduction in sensing gain (i.e., decreased image contrast), thereby degrading environmental sensing performance. In such cases, appropriately reducing the camera’s exposure time effectively increases the bandwidth of the sensing channel, facilitating the recognition of environmental features in dynamic scenes. These results show that the communication and sensing are reciprocal in OC-ISAC, and the camera’s exposure effect has a consistent impact on both functions.

### 5.4. Optimization of Exposure Time

**Figure 12:**
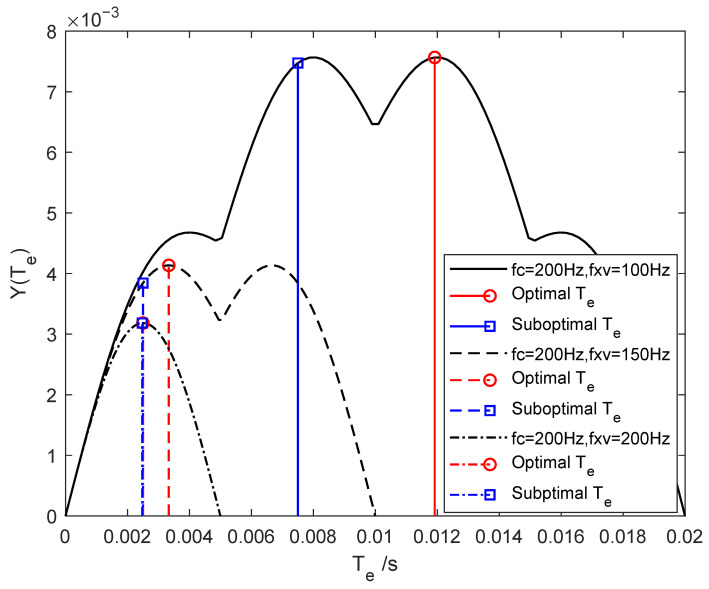
Optimal and suboptimal numerical solutions of the objective function for different exposure times under communication service at $f_{c}=200$ Hz and sensing services at $f_{x}v=$ 100, 150, and 200 Hz.

When communication and sensing coexist, selecting an appropriate camera exposure time is crucial for optimizing both performances. Typically, the communication rate $f_{c}$ exceeds the environmental variation frequency $f_{x}v$ (i.e., $f_{c}>f_{x}v$). Figure 12 shows the objective function in Equation (18) versus exposure time for $f_{c}=200$ Hz and $f_{x}v$ values of 100 Hz, 150 Hz, and 200 Hz. A larger frequency gap (e.g., high-speed communication in a static environment) shifts the optimal exposure time to higher values, making joint optimization more challenging. Conversely, a smaller frequency gap (e.g., low-speed communication or high-speed communication under mobility) leads to lower optimal exposure times, where the requirements of both tasks become more aligned and easier to satisfy. The comparison between square- and circle-marked curves shows that the proposed suboptimal solution closely approximates the global optimum by numerical search, confirming its feasibility.

**Figure 13:**
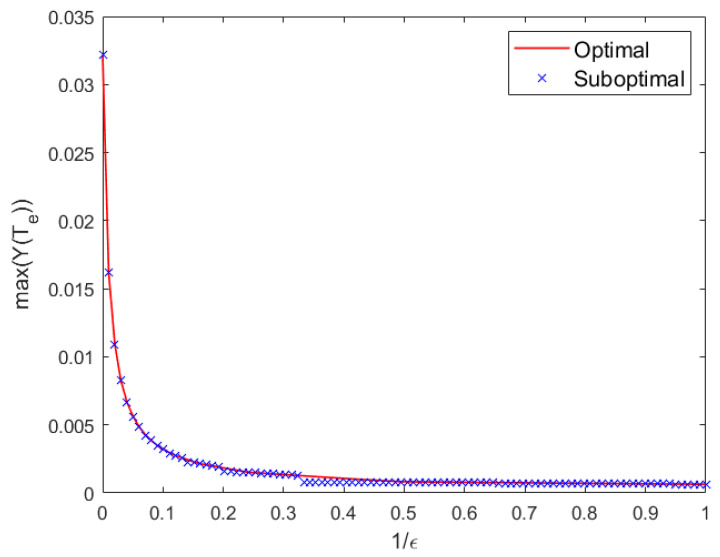
Objective function values achieved with optimal and suboptimal exposure times under different frequency offsets.

To evaluate the effectiveness of the proposed suboptimal algorithm, we set $f_{\min}=f_{x}v$ and $f_{\max}=f_{c}$. Using the sensing frequency $f_{x}v$=100 Hz as a reference, we uniformly sample 1/ϵ over the interval (0, 1] at 100 discrete points to determine the communication frequency according to Equation (20). For each frequency ratio, the optimal exposure time ${\hat{T}}_{e}$ is obtained via numerical search within the feasible range (0, $1/f_{x}v$], while the suboptimal solution ${\tilde{T}}_{e}$ is computed analytically using the proposed algorithm. The corresponding values of the objective function for both solutions are plotted in Figure 13. The normalized deviation (defined as the relative loss in objective function value with respect to the optimum) is on average only 5.71% across the 100 frequency ratios, with a minimum near 0 and a maximum of 36.62%. This close agreement validates the efficacy of the suboptimal approach. The performance gap arises because the suboptimal method places the high-frequency communication component at the center of a sidelobe of the Sinc-shaped frequency response, which is a practical heuristic that, while not always globally optimal, enables closed-form computation and real-time implementation.

## 6. Conclusions

This work theoretically demonstrates the feasibility of integrating communication and sensing using visible-light image sensors within a unified OC-ISAC framework. The analysis identifies camera exposure time as a critical parameter governing the trade-off between communication and sensing performance. Specifically, the exposure-induced low-pass filtering effect distorts the captured pixel response relative to the true incident light intensity variations—whether caused by data-modulated source flickering or environmental motion. Consequently, practical implementations of OCC and vision-based sensing must explicitly account for this effect and employ optimized exposure settings to jointly support both functionalities. The proposed suboptimal exposure time optimization algorithm achieves an excellent balance between performance and computational efficiency. By leveraging a closed-form analytical solution, it reduces complexity from O(N), required by numerical search, to O(1), while incurring only a modest average normalized deviation of 5.71% relative to the global optimum. This minor performance loss is well justified in practical vehicular scenarios, given the priority of real-time responsiveness and low latency. Building on these findings, future research can pursue several promising directions. (1) Adaptive exposure control for OC-ISAC systems that dynamically adjusts exposure time in response to varying data rate demands and vehicle mobility. (2) Robust multi-ROI detection and tracking, enabling reliable maintenance of multiple OCC links through accurate localization and tracking of multiple light sources under heterogeneous ambient lighting conditions. (3) Multi-node vehicular networks based on OC-ISAC, facilitating cooperative sensing and communication among vehicles and infrastructure to enhance situational awareness, network intelligence, and road safety. This work advances the foundational understanding of optical camera-based integrated sensing and communication, positioning cameras not merely as passive sensors but as active, dual-functional nodes in next-generation intelligent transportation systems.

## Figures and Tables

**Table 1:** List of symbols.

Symbol	Description	Unit
*A*	Conversion gain of the image sensor	-
β(d)	Signal attenuation for light propagation over distance *d*	-
$T_{e}$	Exposure time of image sensor	μs
$\vec{r}$	Position vector in 3-D space	m
$I_{c}$	Maximum intensity of the communication light source	
$f_{c}$, $a_{c}$, $\phi_{c}$	Amplitude, frequency, and phase of the communication carrier	V, Hz, rad
$I_{e}$	Maximum intensity of the sensing light source	
$f_{x}$, $\phi_{x}$	Spatial frequency and phase of sensing along given direction *x*	Hz, rad
*v*	Speed of motion	m/s
η	Luminous factor caused by data modulation	-
ξ	Reflective factor caused by environmental mobility	-

**Table 2:** Experimental parameters for the OCC testbed.

Parameter	Unit	Value
Image sensor	-	SONY IM291 (Shenzhen, China)
Image resolution, W×N	-	1080×720
Frame rate	FPS	30
Readout time, $T_{r}$	μs	22.2
Exposure time, $T_{e}$	μs	622, 1202, 2404, 4981
Camera distance	m	0.3
Annular grating diameter, $D_{r}$	m	0.15
Turntable rotation speed, $N_{r}$	rpm	223
Communication frequency, $f_{c}$	Hz	0, 200, 500, 700
Grating width	m	0.01, 0.005, 0.002
Environmental complexity, $f_{x}$	m^−1^	100, 200, 500

## Data Availability

Data underlying the results presented in this paper are not publicly available at this time but may be obtained from the authors upon reasonable request.
